# Kagami Ogata syndrome: a small deletion refines critical region for imprinting

**DOI:** 10.1038/s41525-023-00389-2

**Published:** 2024-01-11

**Authors:** Gonench Kilich, Kelly Hassey, Edward M. Behrens, Marni Falk, Adeline Vanderver, Daniel J. Rader, Patrick J. Cahill, Anna Raper, Zhe Zhang, Dawn Westerfer, Tanaya Jadhav, Laura Conlin, Kosuke Izumi, Ramakrishnan Rajagopalan, Kathleen E. Sullivan, UDN Consortium

**Affiliations:** 1 Division of Allergy and Immunology, The Children’s Hospital of Philadelphia, Philadelphia, PA USA; 2 Division of Rheumatology, The Children’s Hospital of Philadelphia, Perelman School of Medicine at the University of Pennsylvania, Philadelphia, PA USA; 3 Mitochondrial Medicine Frontier Program, Division of Human Genetics, Department of Pediatrics, Children’s Hospital of Philadelphia, and Department of Pediatrics, University of Pennsylvania Perelman School of Medicine, Philadelphia, PA USA; 4 Division of Neurology, Children’s Hospital of Philadelphia and Department of Neurology, Perelman School of Medicine, University of Pennsylvania, Philadelphia, PA USA; 5 Departments of Medicine, Pediatrics and Genetics, Perelman School of Medicine and Children’s Hospital of Philadelphia, Philadelphia, PA USA; 6 Division of Orthopedic Surgery, The Children’s Hospital of Philadelphia, Philadelphia, PA USA; 7 Division of Translational Medicine and Human Genetics, Department of Medicine, Hospital of the University of Pennsylvania, Philadelphia, PA USA; 8 The Center for Biomedical Informatics, The Children’s Hospital of Philadelphia, Philadelphia, PA USA; 9 Division of Genomic Diagnostics, Department of Pathology and Laboratory Medicine, The Children’s Hospital of Philadelphia, Philadelphia, PA USA; 10 Division of Human Genetics, The Children’s Hospital of Philadelphia, Philadelphia, PA USA; 11 Division of Genomic Diagnostics, Department of Pathology and Laboratory Medicine, The Children’s Hospital of Philadelphia, and Department of Pathology and Laboratory Medicine, Perelman School of Medicine, University of Pennsylvania, Philadelphia, PA USA; 12 Present Address: Division of Genetics and Metabolism, Department of Pediatrics, University of Texas Southwestern Medical Center, Dallas, USA

**Keywords:** Genetic testing, Genome

## Abstract

Kagami–Ogata syndrome is a rare imprinting disorder and its phenotypic overlap with multiple different etiologies hampers diagnosis. Genetic etiologies include paternal uniparental isodisomy (upd(14)pat), maternal allele deletions of differentially methylated regions (DMR) in 14q32.2 or pure primary epimutations. We report a patient with Kagami–Ogata syndrome and an atypical diagnostic odyssey with several negative standard-of-care genetic tests followed by epigenetic testing using methylation microarray and a targeted analysis of whole-genome sequencing to reveal a 203 bp deletion involving the *MEG3* transcript and *MEG3:*TSS-DMR. Long-read sequencing enabled the simultaneous detection of the deletion, phasing, and biallelic hypermethylation of the *MEG3:*TSS-DMR region in a single assay. This case highlights the challenges in the sequential genetic testing paradigm, the utility of long-read sequencing as a single comprehensive diagnostic assay, and the smallest reported deletion causing Kagami–Ogata syndrome allowing important insights into the mechanism of imprinting effects at this locus.

## Introduction

Kagami–Ogata syndrome (KOS) is a rare imprinting disorder of 14q32.2 with an estimated incidence of less than 1 in 1,000,000 liveborn individuals. Over 80 cases of KOS have been reported in the literature with a characteristic phenotype including full cheeks with a protruding philtrum, increased angulation of the ribs, and developmental delay/intellectual disability^1^. Imprinting disorders are due to changes in the epigenetic phenomena that allow gene expression predominantly from a single parental allele^2^. Imprinted genes typically form a cluster with differentially methylated regions (DMRs) which regulate expression of the imprinted genes in a parent-of-origin-dependent manner. There are more than 70 imprinting-associated DMRs and each locus has unique transcriptional regulation impacted by imprinting. The human 14q32 imprinting region consists of maternally expressed non-protein-coding genes and paternally expressed protein-coding genes^3,4^. The maternally expressed genes (MEGs) include two long noncoding RNAs: *MEG3* and *MEG8*. The paternally expressed genes include *DLK1* and *RTL1*. There are two DMRs in the 14q32 imprinted region: intergenic DMR (*MEG3/DLK1:*IG*-*DMR) and *MEG3:*TSS-DMR in the promoter region of *MEG3* (Supplemental figure).

Inherited and presumed non-inherited mechanisms can cause imprinting diseases at this locus. *MEG3/DLK1:*IG*-*DMR maintains the parent-of-origin-dependent methylation patterns in the body and placenta, while *MEG3*:TSS-DMR does so only in the body^3,4^. KOS can be caused by paternal uniparental disomy of chromosome 14, maternal deletion of the 14q32.3 imprinted region, or an epimutation with hypermethylation of the DMRs^5^. In contrast, maternal uniparental disomy of chromosome 14, paternal deletion of the 14q32.2 imprinted region, or hypomethylation of DMRs cause Temple syndrome which has a distinct phenotype of growth failure, hypotonia, small hands and feet, broad forehead, high arched palate, and premature puberty thus resembling Prader–Willi syndrome and Silver–Russell Syndrome^6^. The mechanism of disease has important considerations for recurrence risk in future pregnancies.

Here, we report a patient with KOS who was initially diagnosed as having Jeune syndrome and later enrolled in the Undiagnosed Disease Network (UDN). We describe her long diagnostic odyssey including a series of targeted and genome-wide genetic tests, including a negative whole-exome sequencing (WES) and whole-genome sequencing (WGS). A targeted analysis of WGS revealed a de novo 203-bp deletion of the *MEG3* transcript and promoter region. Long-read sequencing of the proband enabled the simultaneous detection of the deletion, phasing of the de novo variant, and the methylation status of the *MEG3:*TSS-DMR locus. The deletion impacts *MEG3* expression and demonstrates that loss of *MEG3* transcription from the maternal allele leads to hypermethylation of *MEG3/DLK1:*IG*-*DMR and *MEG3:*TSS*-*DMR. This small deletion refines our understanding of the imprinting mechanism in Kagami–Ogata syndrome.

## Results

### Case report

A 9-month-old girl presented to the UDN with features felt to be due to Jeune syndrome. She was conceived via in vitro fertilization (IVF) and was born at 31 weeks and 4 days gestation due to premature rupture of the membranes to a 31-year-old gravida 2, para 1 mother. The delivery was vaginal, vertex, and uncomplicated. Polyhydramnios occurred during pregnancy and a 20-week prenatal ultrasound showed fetal hepatosplenomegaly, ventriculomegaly, total body edema, thickened nuchal fold and echogenic kidneys. Amniocentesis was performed for microarray, karyotype, and fluorescence in situ hybridization (FISH) for trisomy 13, 18, and 21 which were all normal.

**Fig. 1:**
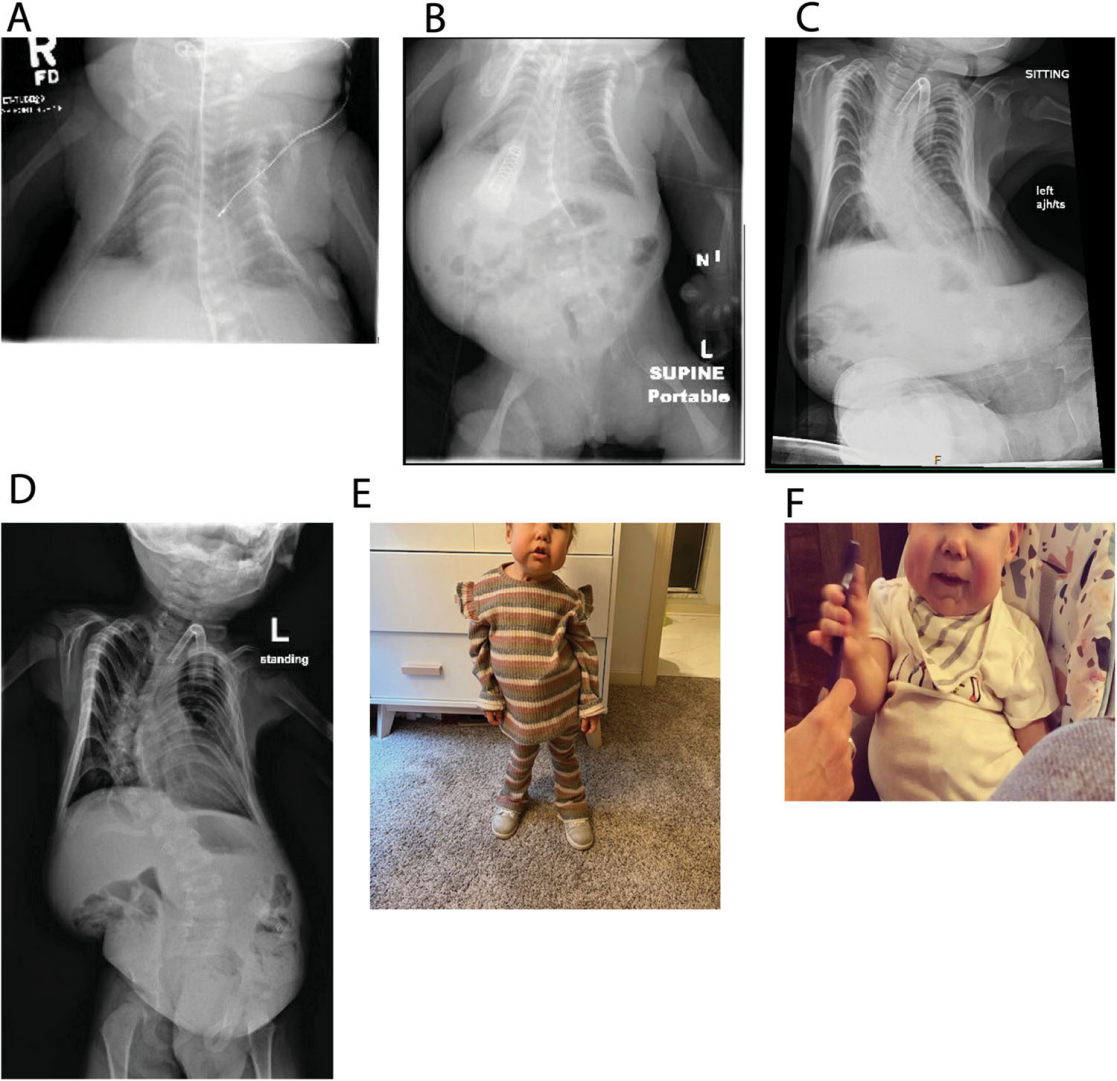
Clinical features of the patient. **A** Shortly after birth, the “coat hanger” rib deformities are seen. **B** Supine view demonstrating the impact of her rib cage and scoliosis on her abdominal content. **C** A seated view of the impact of the rib cage and scoliosis on her abdominal contents. **D** The acetabular dysplasia is seen on this view and the progression of the scoliosis. **E** Toddler and **F** infant views of the child. Her full cheeks and small chin can be observed. The patient’s family consented to publication of these photographs.

The Apgar scores were 6, 1, and 8 at 1, 5, and 10 minutes of life respectively. The infant required positive-pressure ventilation for cyanosis and apnea. She was noted to have extensive lymphedema, dysmorphic features, and hypotonia on initial exam. Chest X-rays showed a severe bell-shaped chest, handle-bar clavicles, thin ribs, and left lung atelectasis. Abdominal X-ray was notable for hepatomegaly, bilateral acetabular dysplasia, and flaring of iliac wings (Fig. 1). Initial cranial ultrasound was unremarkable except for extensive scalp edema and a spinal ultrasound was concerning for a tethered cord. Cardiac echocardiogram showed patent foramen ovale (PFO), patent ductus arteriosus (PDA), mild right atrial enlargement and a mildly dilated right ventricle with normal systolic wall motion. She passed her newborn hearing test at 3 months old.

She was admitted to the NICU for 3 months. On the 45th day of life she underwent surgery for placement of a tracheostomy for respiratory failure and gastrostomy tube for dysphagia. Her neonatal course was further complicated by non-ketotic hypoglycemia, hyperinsulinemia, nephrotic syndrome, and MRSA bacteremia and tracheitis. An immune evaluation was performed due to her infections and was normal. After discharge, she was ventilator-dependent for another 6 months. Her neonatal problems resolved, and she started receiving Early Intervention Services: physical therapy, speech therapy, occupational therapy and feeding therapy; with good progress.

Physical exam findings were significant for small chin, low set/posteriorly rotated ears, narrow and bell-shaped chest, rhizomelic appearing limbs, ulnar deviation at wrists, bent 3rd finger on left hand, hepatomegaly, contractures of extensor tendons on dorsum of feet, mild scaphocephaly, horizontal nystagmus, intermittent R > L esotropia, hypertelorism and shallow coccygeal dimple (Fig. 1). There was concern for increasing head circumference and a brain MRI showed ventriculomegaly involving lateral and third ventricles. She also was found to have collapsed long thoracolumbar scoliosis measuring over 50 degrees when she was sitting. Our patient had some features consistent with KOS^1^ (Supplemental Table) although her rib features were more severe than most published cases.

She is now 46 months old and was decannulated at 27 months of age. Her bell-shaped chest and respiratory status have improved. Her scoliosis continues to progress and is in the moderate range. She is developmentally delayed in gross and fine motor skills but has been walking independently since 20 months of age and said her first words at 12 months of age. Her receptive language is thought to be normal. She is making progress with oral feeds even though she is still dependent on G-tube feeds. After KOS was diagnosed, her medical regimen has been simplified. She recently had a normal abdominal ultrasound and serum α-fetoprotein level of 3 (<20 ng/mL) and will be regularly screened for hepatoblastoma, an unquantified risk in KOS.

### Diagnostic evaluations

**Fig. 2:**
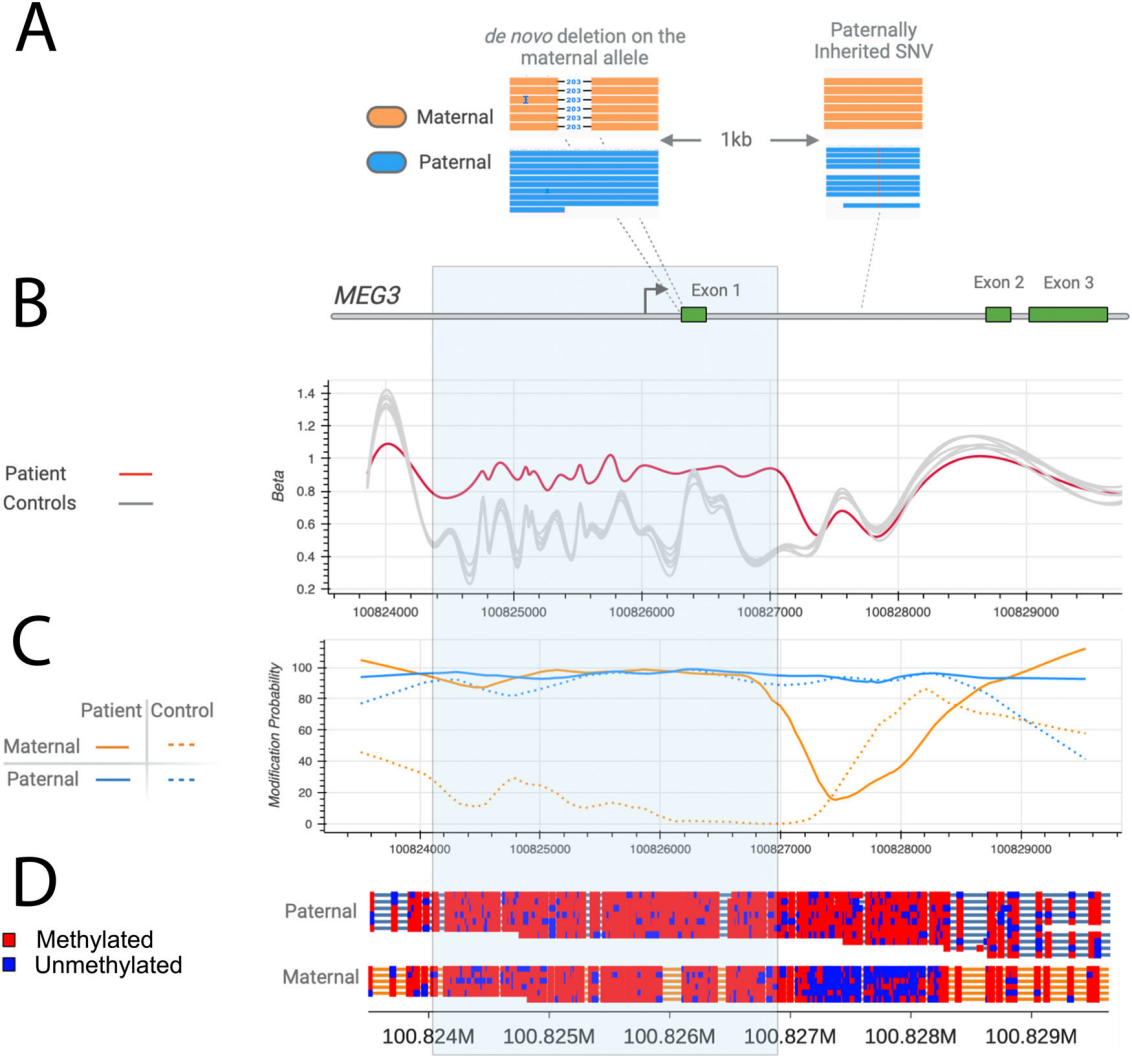
Long-read sequencing. **A** A schematic diagram of the deletion revealed though long-read sequencing. The paternal variant is shown in the right-hand section which allowed phasing of the de novo deletion, which is shown in the left block of reads on the maternal allele. Long-reads were phased using heterozygous variants in the genome and the maternal/paternal haplotypes were differentiated using the previously existing parental WGS data. **B** The methylation profiles from Illumina Epic 850k array. The proband is the red tracing and six controls from a publicly available dataset (GSE153211) are shown in gray. **C** The phased methylation probabilities identified from PacBio data. The solid lines show the patient data while an internal control is shown as dashed lines. The patient data shows the hypermethylation of maternal allele confirming the diagnosis of Kagami–Ogata syndrome. **D** Visualization of methylation probabilities along the reads in the *MEG3:*TSS-DMR region. Each line represents a read and regions shaded in red are methylated while blue are unmethylated. Unshaded regions denote the lack of methylation data. CpG islands in the region are displayed as green horizontal bars.

**Fig. 3:**
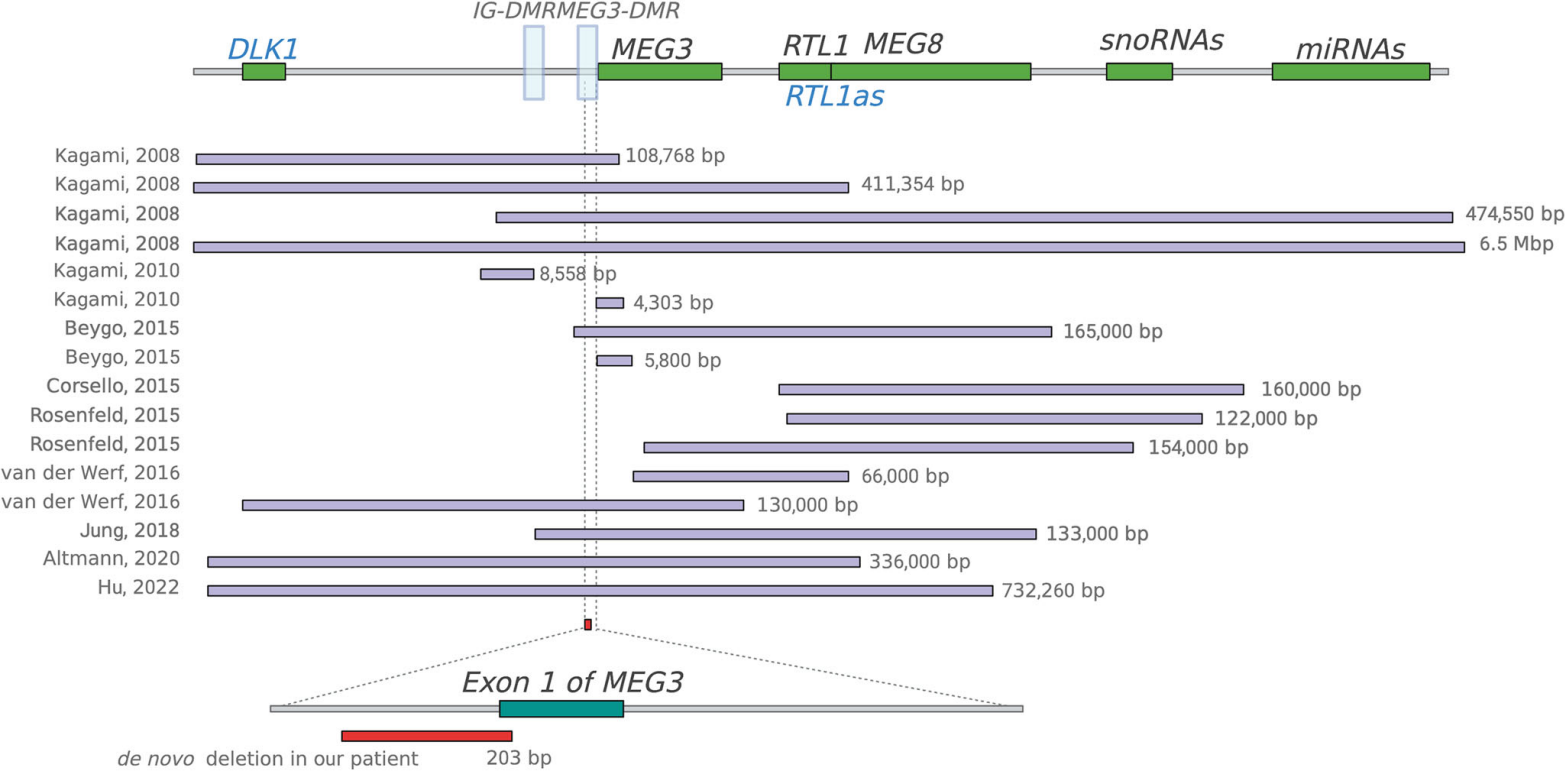
Genomic locations and sizes of deletions causing Kagami–Ogata syndrome. A comparison of the 203 bp de novo deletion in our patient is shown in red (hg38 chr14: 100825921-100826123/NC_000014.9:g.100825921_100826123del) and previously reported deletions causing Kagami–Ogata syndrome. Paternally expressed genes are labeled in blue. The length of each deletion is specified on the right.

The patient was seen in the UDN at 24 months of age. Previous post-natal genetic testing included a non-diagnostic skeletal dysplasia panel (Invitae Skeletal Dysplasia Panel) with multiple variants of uncertain significance in 5 different genes (none felt to be candidates), a negative trio WES (GeneDx XomeDxPlus), negative mitochondrial DNA analysis (GeneDx), and a negative deletion/duplication analysis of skeletal dysplasia genes (Custom testing at Division of Medical Genetics, UPMC Children’s Hospital of Pittsburgh). The reanalysis of WES data by the UDN team was non-diagnostic. Upon review by the UDN team, including an ad hoc member specializing in thoracic dystrophy, we concluded that thoracic dystrophy was unlikely, and we expanded our testing to WGS and DNA methylation. The WGS official report was negative but commercial testing of DNA methylation (Greenwood Genetic Center) was abnormal suggesting a methylation defect. Reanalysis of WGS on site revealed a 203 bp de novo deletion (hg38 chr14: 100825924-100826126/NC_000014.9:g.100825924_100826126del) overlapping the *MEG3* transcript and the promoter region which was later confirmed by long-read sequencing (Figs. 2 and 3). *MEG3* is a noncoding RNA and is typically not covered in WES. The small deletion was not reported on the WGS report as it was below the resolution of the reportable deletions (typically about 3 kb). The short-read sequencing could not establish the parent-of-origin of the 203 bp deletion, therefore long-read sequencing was performed. The *MEG3* region was phased using informative heterozygous SNVs (read-based phasing using *whatshap*) and the parental origin of the two haplotypes were determined using the nearest paternally inherited SNV revealing the deletion on the maternal allele. Methylation profiling using long-read sequencing also revealed that the DNA methylation of the maternal allele was also aberrant. With this information, we established the diagnosis of KOS in this patient, determined that the deletion was apparently de novo, and based on the etiology were able to provide the family with important recurrence risk information.

## Discussion

The patient reported here exemplifies many of the pitfalls in establishing a correct diagnosis. Physicians anchored on her chest phenotype, leading to a tentative diagnosis of Jeune syndrome and delaying more genome-wide approaches. Standard-of-care WES and WGS analyses failed to identify the 203 bp deletion involving the promoter region which extended to the exon 1 of *MEG3* highlighting the analytic challenges. The *MEG3* gene is a noncoding RNA and typical exome sequencing assays may not include baits targeting noncoding RNAs. Single exon copy-number variants are challenging to detect using exome sequencing and first exons are even more challenging due to the high GC content. Similarly, the size of this deletion (203 bp) makes it very challenging to detect with WGS as well. Most of the algorithms to detect copy number variants from WGS use mean depth of coverage across evenly divided bins of a certain size (typically 1 to 5 kb) and a 203 bp deletion is under the reporting limits of most of the read-depth based copy number algorithms. The structural variant callers that use split-read alignments can potentially detect a deletion of this size, but they are known to be enriched for false positives making the prioritization of this variant extremely challenging. Long-read sequencing has several advantages compared to the short-read sequencing assays including WES and WGS. Long-read sequencing can detect all major classes of variants in a more comprehensive manner and methylation with phasing in a single assay. In this patient, long-read sequencing established that the deletion was on the maternal allele, found the aberrant methylation at this locus and confirmed the diagnosis in a single experiment. While long-read sequencing has the potential to become the single most comprehensive assay, some challenges remain. It is challenging to perform a hypothesis free, genome-wide analysis using long-read sequencing data as it has some of the same analytical challenges as WGS short-read sequencing data analysis and lacks resources such as large population scale databases which may aid in variant prioritization (e.g., gnomAD). While it is possible to phase and differentiate two haplotypes using informative heterozygous SNVs, determining the parent-of-origin requires informative parental variants SNVs or known imprinting signatures.

The prognosis of KOS reflects the respiratory status primarily and most reported deaths occur early in life^1^. Several patients have had hepatoblastoma and one died of hemophagocytic syndrome while under treatment for hepatoblastoma, however, the true frequency of hepatoblastoma in KOS is not known. Thus, current recommendations are to screen with alpha fetoprotein levels every three months until 4 years of age^1^. The child is currently doing well but faces surgery for scoliosis.

Maternal allele deletions have been recognized as causal for KOS since 2010^4^ with the smallest previous reported size of 5.8 kb for disease^3^. Mapping of a nested deletion series led to the hypothesis of a hierarchy of effects where the *MEG3/DLK1:*IG*-*DMR was presumed to regulate the *MEG3:*TSS-DMR methylation on the maternal allele^3^. Expression of *MEG3* is downregulated by EZH2, HDAC1, and DNMT1 in cancer suggesting that *MEG3* expression is downstream of the DMRs post-embryologically^7^. In stem cells, *MEG3* regulates EZH2-mediated repression of DLK1 on the maternal allele^8^. There are additional miRNAs and snoRNAs downstream of *MEG3* which have been hypothesized to contribute overall to imprinting and this deletion does not encompass them, further isolating the role of *MEG3*^9^ (Supplemental Figure). The structure of the *MEG3* transcript is important and the GC content drives the highly complex structure felt to be important in protein binding^10^. Many of the effects are presumed to be driven during early development where promoters marked with H3K27me3 gain DNA methylation during embryologic differentiation^11–13^. Our patient’s small deletion impacting the *MEG3* promoter and transcript demonstrates that *MEG3* transcripts are required to prevent methylation of both the *MEG3/DLK1:*IG*-*DMR and *MEG3:*TSS-DMR and suggests that the snoRNAs and miRNAs at this locus have a much lesser role in DMR methylation and in the clinical phenotype.

This case was instructive due to the very small deletion, essentially comprising the promoter and proximal exon of *MEG3*. Imprinting disorders are due to changes in the epigenome either secondary to DNA mutations in a *cis* or *trans* arrangement or primary epimutations in the absence of recognized DNA sequence changes^2^. Although epimutations are heritable in subsequent cell divisions, they are not heritable across generations and thus have a distinct inheritance pattern. The distinction between the possible etiologies of imprinting disorders has important implications for the families. In spite of great effort to establish a diagnosis using clinically available testing, this child remained without a definitive diagnosis, hindering assessments of prognosis, and impairing medical decision making regarding best treatments. Our finding of a small deletion impacting *MEG3* transcription is important because lack of identification would have led to improper recurrence risk assessment. This also raises the concern that some patients felt to have epimutations might have very small or even single nucleotide variants hypothetically that could be heritable as causes of KOS.

## Methods

### Ethics

The patient’s family self-enrolled in the UDN and consented to evaluation and sharing of results. This program is NIH-funded and was reviewed by the NIH Institutional Review Board. The child’s parents provided written informed consent for her participation and their own participation. The authors affirm that human research participants provided informed consent, for publication of the images in Fig. 1 and provided consent to publish.

### Molecular genetic analysis

DNA on the proband and parents was extracted from whole blood, as per UDN protocol (Promega blood DNA kit AS1400 on the Promega Maxwell instrument, Promega, Madison, WI). Aliquots were used for unbiased DNA methylation analysis (EpiSign, Greenwood Genetic Center), WGS, and long-read sequencing. The WGS was performed at Baylor Human Genome Sequencing Center using a PCR-free library preparation with 500 bp insert size according to the KAPA Hyper Prep kit (Roche, Indianapolis, IN). Sequencing was performed on Illumina Novaseq 6000 platform for 150 bp paired end reads to achieve a minimum average coverage of 60x. Short-read sequencing data was aligned using BWA and GATK best practices were used to produce variant calls^14^.

PacBio long-read sequencing was performed at the Genomics Core Facility at Icahn School of Medicine using Sequel IIe platform using three SMRTcells to achieve 20x mean coverage across the genome. Raw PacBio sequence reads were aligned to the reference genome hg38 using pbmm2^15^, and the DeepVariant^16^ variant caller was used to detect single nucleotide variants and small insertion deletions. The pbsv software tool^17^ was used to call structural variants and whatshap^18^ was used to phase variants using informative genotypes. Methylation probabilities were computed using ccsmeth/CpG-tools^19–21^ and visualized using modbamtools and IGV^22,23^. A targeted analysis of *MEG3* locus was performed in both short-read and long-read sequencing data. Parental short-read sequencing data was used to determine the parent-of-origin of the apparently de novo variant in the proband.

### Reporting summary

Further information on research design is available in the Nature Research Reporting Summary linked to this article.

### Supplementary information

Supplemental Material

Reporting Summary

## Data Availability

Data is available upon request to the corresponding author and has been deposited in dbGaP (see below). Accession numbers for sequence data are: Phenome Central: P0013787 (https://www.phenomecentral.org/). dbGaP: phs001232.V5.p2 (https://www.ncbi.nlm.nih.gov/gap/).
