# Supplementary material for: Kagami Ogata syndrome: a small deletion refines critical region for imprinting
Source: NPJ Genom Med. 2024 Jan 11;9:5. doi: 10.1038/s41525-023-00389-2 (PMC10784583; doi:10.1038/s41525-023-00389-2)
Supplement: Supplementary file 1 — Supplemental Material [file 41525_2023_389_MOESM1_ESM.docx]

**Supplemental information**

**Undiagnosed Disease Network collaborators**

Undiagnosed Diseases Network: Margaret Adam, Raquel L Alvarez, Justin Alvey, Laura Amendola, Ashley Andrews, Euan A Ashley, Mahshid S Azamian, Carlos A Bacino, Guney Bademci, Ashok Balasubramanyam, Dustin Baldridge, Jim Bale, Michael Bamshad, Deborah Barbouth, Pinar Bayrak-Toydemir, Anita Beck, Alan H Beggs, Edward Behrens, Gill Bejerano, Hugo J Bellen, Jimmy Bennett, Beverly Berg-Rood, Jonathan A Bernstein, Gerard T Berry, Anna Bican, Stephanie Bivona, Elizabeth Blue, John Bohnsack, Devon Bonner, Lorenzo Botto, Brenna Boyd, Lauren C Briere, Elly Brokamp, Gabrielle Brown, Elizabeth A Burke, Lindsay C Burrage, Manish J Butte, Peter Byers, William E Byrd, John Carey, Olveen Carrasquillo, Thomas Cassini, Ta Chen Peter Chang, Sirisak Chanprasert, Hsiao-Tuan Chao, Gary D Clark, Terra R Coakley, Laurel A Cobban, Joy D Cogan, Matthew Coggins, F Sessions Cole, Heather A Colley, Cynthia M Cooper, Heidi Cope, William J Craigen, Andrew B Crouse, Michael Cunningham, Precilla D'Souza, Hongzheng Dai, Surendra Dasari, Joie Davis, Jyoti G Dayal, Esteban C Dell'Angelica, Katrina Dipple, Daniel Doherty, Naghmeh Dorrani, Argenia L Doss, Emilie D Douine, Laura Duncan, Dawn Earl, David J Eckstein, Lisa T Emrick, Christine M Eng, Cecilia Esteves, Marni Falk, Elizabeth L Fieg, Paul G Fisher, Brent L Fogel, Irman Forghani, Ian Glass, Bernadette Gochuico, Page C Goddard, Rena A Godfrey, Katie Golden-Grant, Alana Grajewski, Irma Gutierrez, Don Hadley, Sihoun Hahn, Meghan C Halley, Rizwan Hamid, Nichole Hayes, Frances High, Anne Hing, Fuki M Hisama, Ingrid A Holm, Jason Hom, Martha Horike-Pyne, Alden Huang, Sarah Hutchison, Wendy J Introne, Rosario Isasi, Fariha Jamal, Gail P Jarvik, Jeffrey Jarvik, Suman Jayadev, Orpa Jean-Marie, Vaidehi Jobanputra, Lefkothea Karaviti, Jennifer Kennedy, Shamika Ketkar, Dana Kiley, Shilpa N Kobren, Isaac S Kohane, Jennefer N Kohler, Susan Korrick, Mary Kozuira, Deborah Krakow, Donna M Krasnewich, Elijah Kravets, Seema R Lalani, Byron Lam, Christina Lam, Brendan C Lanpher, Ian R Lanza, Kimberly LeBlanc, Brendan H Lee, Roy Levitt, Richard A Lewis, Pengfei Liu, Xue Zhong Liu, Nicola Longo, Sandra K Loo, Joseph Loscalzo, Richard L Maas, Calum A MacRae, Valerie V Maduro, Rachel Mahoney, Bryan C Mak, Laura A Mamounas, Teri A Manolio, Rong Mao, Kenneth Maravilla, Ronit Marom, Gabor Marth, Beth A Martin, Martin G Martin, Julian A Martínez-Agosto, Shruti Marwaha, Jacob McCauley, Allyn McConkie-Rosell, Alexa T McCray, Elisabeth McGee, Heather Mefford, J Lawrence Merritt, Matthew Might, Ghayda Mirzaa, Eva Morava, Paolo Moretti, Mariko Nakano-Okuno, Stanley F Nelson, John H Newman, Sarah K Nicholas, Deborah Nickerson, Shirley Nieves-Rodriguez, Donna Novacic, Devin Oglesbee, James P Orengo, Laura Pace, Stephen Pak, J Carl Pallais, Christina G S Palmer, Jeanette C Papp, Neil H Parker, John A Phillips 3rd, Jennifer E Posey, Lorraine Potocki, Barbara N Pusey Swerdzewski, Aaron Quinlan, Deepak A Rao, Anna Raper, Wendy Raskind, Genecee Renteria, Chloe M Reuter, Lynette Rives, Amy K Robertson, Lance H Rodan, Jill A Rosenfeld, Natalie Rosenwasser, Francis Rossignol, Maura Ruzhnikov, Ralph Sacco, Jacinda B Sampson, Mario Saporta, Judy Schaechter, Timothy Schedl, Kelly Schoch, Daryl A Scott, C Ron Scott, Vandana Shashi, Jimann Shin, Edwin K Silverman, Janet S Sinsheimer, Kathy Sisco, Edward C Smith, Kevin S Smith, Emily Solem, Lilianna Solnica-Krezel, Benjamin Solomon, Rebecca C Spillmann, Joan M Stoler, Jennifer A Sullivan, Angela Sun, Shirley Sutton, David A Sweetser, Virginia Sybert, Holly K Tabor, Queenie K-G Tan, Amelia L M Tan, Mustafa Tekin, Fred Telischi, Willa Thorson, Camilo Toro, Alyssa A Tran, Rachel A Ungar, Tiina K Urv, Matt Velinder, Dave Viskochil, Tiphanie P Vogel, Colleen E Wahl, Melissa Walker, Stephanie Wallace, Nicole M Walley, Jennifer Wambach, Jijun Wan, Lee-Kai Wang, Michael F Wangler, Patricia A Ward, Daniel Wegner, Monika Weisz Hubshman, Mark Wener, Tara Wenger, Katherine Wesseling Perry, Monte Westerfield, Matthew T Wheeler, Jordan Whitlock, Lynne A Wolfe, Kim Worley, Changrui Xiao, Shinya Yamamoto, John Yang, Stephan Zuchner

**Figure**


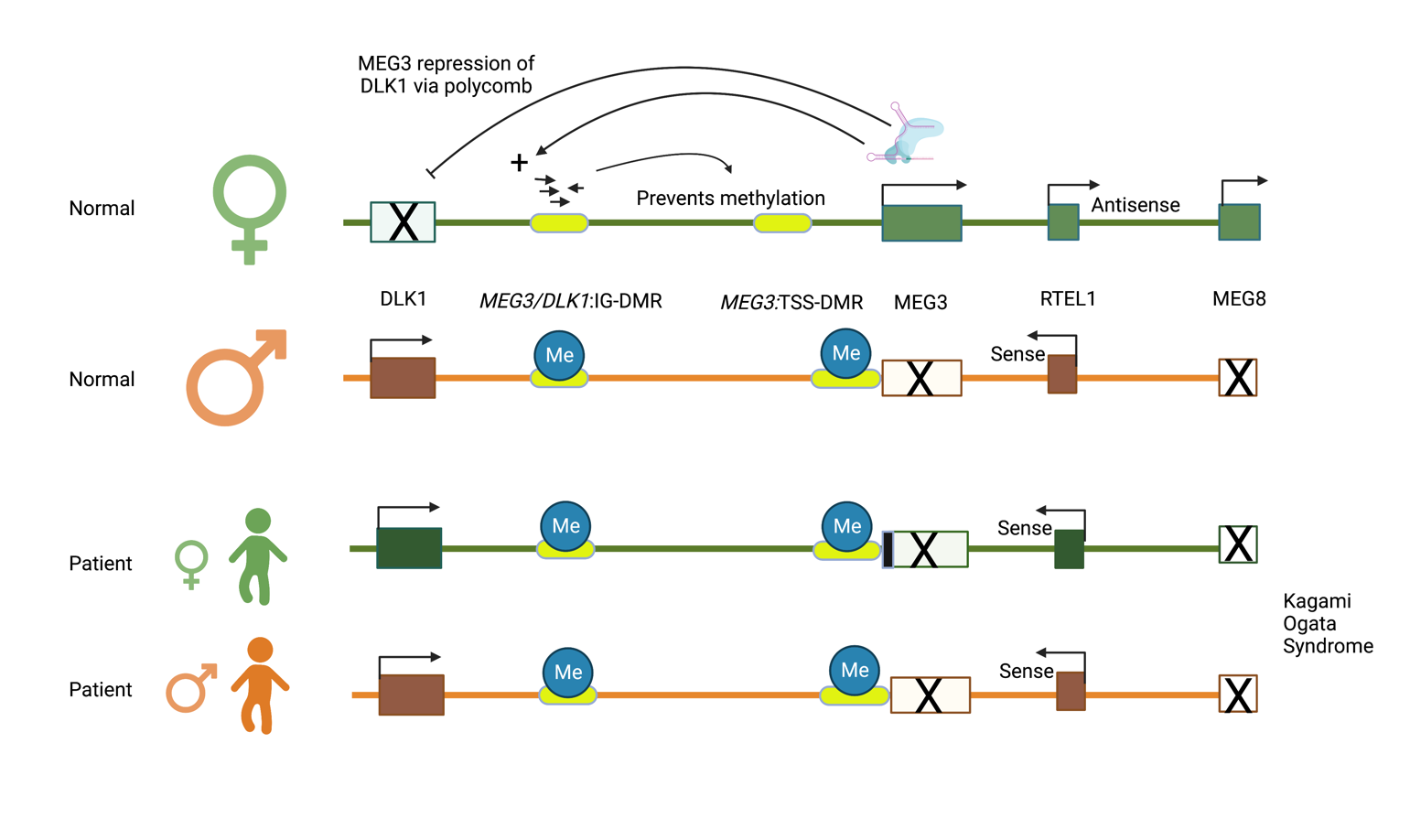


Supplemental figure. Model of *MEG3* deletion effect. The normal maternal and paternal alleles are schematically displayed in the upper two rows. The maternal allele is normally unmethylated at the *MEG3/DLK1*:IG-DMR and *MEG3*:TSS-DMR and the MEG3 transcript itself inhibits methylation. Small non-coding RNAs originating from the *MEG3/DLK1*:IG-DMR are hypothesized to prevent methylation at the MEG3-DMR and to promote expression of *MEG3* transcription, completing the reinforcement loop on the maternal allele. Not shown are the miRNAs and snoRNAs that are embedded through the *MEG3* to *MEG8* region. The paternal allele is methylated at both *MEG3/DLK1*:IG-DMR and *MEG3*:TSS-DMR and therefore does not express *MEG3*, thereby allowing *DLK1* to escape repression by the *MEG3* RNA. *RTEL1* is expressed from both alleles either from a sense or antisense direction. The patient’s deletion is shown as the black vertical line encompassing the edge of the *MEG3*:TSS-DMR and a portion of the first exon of *MEG3*. (Produced in Biorender)

Supplemental Table

**Published clinical and genetic features.**

| **Clinical feature** | **Frequencies reported ^1^** | **Our patient** |
| --- | --- | --- |
| Polyhydramnios | 95% | X |
| Microcephaly | 42% |  |
| Anteverted nares | 45% | X |
| Depressed nasal bridge | 71% | X |
| Small ears | 32% |  |
| Full cheeks | 45% | X |
| Short palpebral fissures | 51% |  |
| Frontal bossing | 45% | X |
| Hirsute forehead | 40% |  |
| Protruding philtrum | 70% |  |
| Puckered lips | 32% |  |
| Micrognathia | 68% |  |
| Short neck | 75% | X |
| Bell-shaped thorax | 90% | X |
| Coat hanger ribs | 85% | X |
| Respiratory insufficiency | 90% | X |
| Hypotonia | 30% | X |
| Developmental delay | 72% | X |
| Feeding difficulties | 70% | X |
| Abdominal wall defects | 90% |  |
| Cardiac disease (ASD, VSD, PDA) | 25% | X |
| Seizures | 7% |  |
| Contractures | 52% |  |
| Kyphoscoliosis | 25% | X |
| Hepatomegaly | 10% | X |
| Hepatoblastoma | 3% |  |
| Inguinal hernia | 18% |  |
| UPD | 60% (24% of which were with RT, and 4% of which were mosaic) |  |
| Microdeletion | 25% | X |
| Epimutation | 9% |  |
| Unknown | 6% |  |

ASD= Atrial septal defect, VSD= Ventricular septal defect, PDA= Patent ductus arteriosus, RT= Robertsonian translocation, UPD= Uniparental disomy
